# Exploring equity, diversity, and inclusion in a simulation program using the SIM-EDI tool: the impact of a reflexive tool for simulation educators

**DOI:** 10.1186/s41077-023-00250-7

**Published:** 2023-03-31

**Authors:** Eve Purdy, Ben Symon, Ruth-Ellen Marks, Chris Speirs, Victoria Brazil

**Affiliations:** 1 Gold Coast Hospital and Health Service, Southport, QLD Australia; 2 Bond University, Faculty of Health Sciences & Medicine, Gold Coast, QLD Australia; 3 Prince Charles Hospital, Brisbane, QLD Australia; 4 Simulation Training on Resuscitation for Kids, Queensland Children’s Hospital, Brisbane, Australia

## Abstract

**Background:**

There have been increasing calls for awareness and action related to equity, diversity, and inclusion (EDI) in simulation but a lack of practical guidance for how simulation delivery teams (SDTs) might move towards meaningful transformation. The gap between academic conversations about EDI and how to practically impact SDT attitudes, behaviors, and performance remains considerable. We designed a conversational tool, the SIM-EDI, to bridge the gap between theory and practice for SDTs by enhancing reflexivity and studied its impact locally.

**Methods:**

We engaged in a collaborative autoethnography to explore EDI within our emergency department SDT shortly after implementing the SIM-EDI. The 12-month ethnography is informed by our team’s collection and analysis of data about ourselves and our own experiences using the tool. Data included serial interviews, field notes from simulations and SDT meetings, SDT documents, and self-reflections.

**Results:**

We found the SIM-EDI tool could be implemented with a team with a high level of readiness. Use of the tool had several meaningful impacts including enhanced team reflexivity, normalization of conversations related to EDI and increased confidence to engage in EDI conversations with participants. Key themes throughout the process included (1) individual and team growth, (2) fear of “getting it wrong”, and (3) tension between bias towards action and need for slow reflection.

**Conclusion:**

The SIM-EDI tool can effectively promote reflexivity among faculty in an emergency department simulation program. The tool is easy to use and implement, impacts attitudes and behaviors, and facilitates individual and team growth.

**Supplementary Information:**

The online version contains supplementary material available at 10.1186/s41077-023-00250-7.

## Introduction

**Fig. 1:**
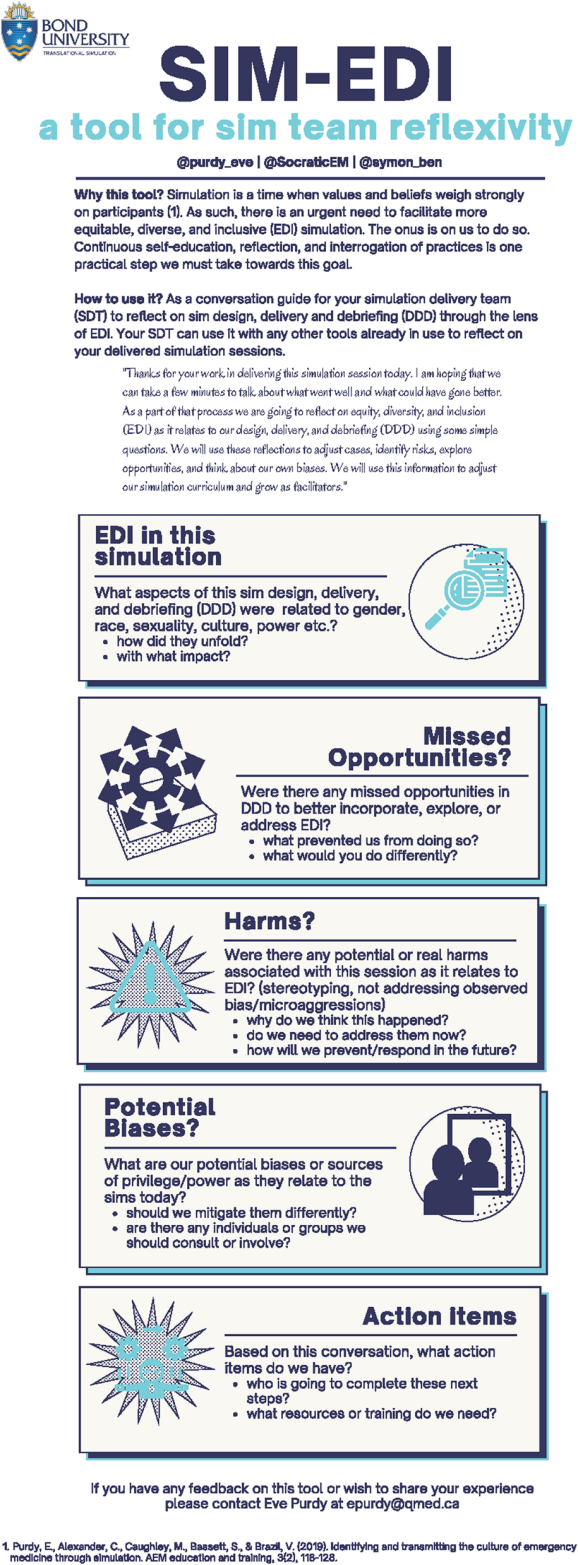
SIM-EDI Tool

There have been increasing calls for awareness and action related to equity, diversity, and inclusion (EDI) within simulation but a lack of practical guidance for how simulation delivery teams (SDTs) might move towards meaningful transformation. Recent reviews have challenged the simulation community to aspire to ideals of cultural humility and criticality [1–3]. The goals are that such perspectives can ethically shape safe learning environments and contribute to a shift in training towards meeting needs of the diverse patient populations we serve. Academic conversations are a first step but are too far removed from concrete action—many practitioners are left wondering, “but what should I actually do?”, while others jump straight into action without clear understanding of potential consequences. The gap between academic conversations about EDI and how to practically impact SDT attitudes, behaviors, and performance remains considerable. Below, we present our team’s experience using a reflective tool, the SIM-EDI (Fig. 1), that we previously designed to bridge the gap between theory and practice as it relates to EDI in simulation. Our findings show the regular use of a reflective tool for SDT teams is possible and our experience provides insights into some issues teams might face when moving towards simulation programs that are more equitable, diverse, and inclusive.

This paper is written specifically for readers who already understand the importance of attending to EDI in simulation and who are looking for ways to shape their practice towards those ideals. We direct readers who are still wondering about why EDI is important to our first four references or more general reading about EDI in education and health outcomes [1–4].

While academic debates about the frameworks for EDI in simulation have been simmering, simulation practice has forged on. The spectrum of these practices—from highly variable efforts to incorporate EDI specific learning objectives to simple business as usual simulation—highlights the potential benefits of a tool to facilitate reflective practice that serves all simulation practitioners. For example, one exemplary group, Nakajima et al., performed an in-depth needs analysis in diverse settings to inform the design of scenarios specific to EDI. They then used a structured method for scenario design including rigorous community consultation [4]. By contrast, another group reported a “mass simulation exercise” (each station complete with only 2 min of feedback) to teach “cultural competencies” related to 8 patient presentations (e.g., mistrust of African American patients in the healthcare system) with seemingly little regard for potential negative consequences of stereotyping or the problematic framing as EDI as a competency that can be achieved [5]. These serve as divergent examples. The contrast highlights dramatic differences in understanding of how EDI specific learning objectives might be thoughtfully incorporated into simulation and ability to mitigate potential risks. For many groups however (including our SDT), there has been less deliberate effort—most have simply been delivering the same simulations they always do. Even so, the everyday decisions in design, delivery, and debriefing have significant implications for EDI within programs—whether SDTs are aware of those ramifications or not. A tool that allows a group to regularly reflect on EDI in their program would allow for ongoing criticality, awareness, and growth regardless of team’s starting point.

Reflexivity—the examination of one’s own beliefs, judgements, and practices—is a necessary precursor to thoughtful action and is well-recognized as essential by feminist and critical race scholars [6, 7]. The centrality of reflexivity to meaningful engagement with topics related to EDI serves as the foundational theoretical underpinning for our approach. We feel strongly that SDTs must habituate the interrogation of practices and prioritize meaningful reflection if they are to move their local needle on EDI in simulation with the authenticity, thoughtfulness, and rigour it deserves. Engaging in informal conversations with colleagues (so called “Kitchen-table reflexivity”) has been identified as one way to deeply engage in understanding positionality and power [8]. We found the simplicity and accessibility of this approach attractive, but we were unable to locate a tool that would practically guide our team through such conversations. To fill this gap, we created and implemented our own tool, the SIM-EDI (Fig. 1). The tool asks simple but pointed questions to promote reflection on one’s own assumptions and how those assumptions impact the simulation and participants. We implemented the tool with our emergency department (ED) SDT over a 12-month period. The current study captures our experience using this tool and its impact on our understanding of our approach to EDI in simulation.

## Methods

### Overview

We engaged in a collaborative autoethnography [9] to explore EDI within our ED SDT shortly after implementing the SIM-EDI. Autoethnography is an approach to research that aims to describe and systematically analyze one’s own personal experiences. Collaborative autoethnography has the same aims but is done in groups to make sense of a collective experience. Collaborative autoethnography has been previously identified as a pathway for transformative learning [10], and we believe that understanding of EDI in the simulation context is constructed through situated social and cultural experiences. For these reasons, collaborative autoethnography was an appropriate methodologic choice. Our collaborative autoethnography is informed by our team’s collection and analysis of data about ourselves and our own experiences. Data included serial interviews, field notes from simulations and SDT meetings, SDT documents, self-reflections, and group reflections.

### Context and participants

Gold Coast University Hospital is a large tertiary care center in Queensland, Australia. The ED education program includes a 2-h simulation session each week attended by 4 emergency medicine registrars and 6–8 nurses delivered by the SDT which is made up of nurse educators, consultants with educational portfolios, simulation/education fellows and registrar, and the simulation team. Participants engage in two scenario-based simulations focused on common and important emergency department presentations, e.g., chest pain, toxicology, trauma, geriatric care, unwell children, and acute behavioral disturbance. Patients are manifested either by mannikins or simulated patient actors. Each patient care episode is followed by a 20-min debrief to reflect on individual and group learning. The focus is on teamwork and systems-based practice. After each simulation session, the SDT meets to discuss the simulation design, delivery, and debriefing.

All of the ED SDT (12 total) were invited to be collaborators in this study and 10 decided to participate. The group currently has multiple degrees of diversity including gender, LGBTQ2+, religious, nationality, language, and professional background. Notably, at this moment in time, we have limited visible racial diversity in our group.

This study was approved by the Gold Coast University Hospital Research Ethics Committee (LNR/2021/QGC/77284).

### Data collection and management

Data collection for this collaborative autoethnography took different forms including interviews, field notes from SDT team reflections, and self-reflections. See Table 1 for more details about these methods. All audio content (interviews and SDT team reflection audio field notes) were transcribed using Otter AI transcription software then made de-identifiable [11]. All data was input to NVivo 12 for analysis [12].

**Table 1:** Methods of Data Collection

**Data source**	**Details**
Interviews	For each participant, two interviews were conducted. One was conducted within the first week of the study period and one ~ 6–9 months of using the facilitated reflection tool. In collaborative ethnography, it is common for participants to interview each other. In this case, VB/EP conducted the initial interviews; however, participants did have the option to be interviewed by an external interviewer BS. BS conducted all the interviews at the 9-month time-period. The semi-structured interview guide is available in Additional file 1
SDT team reflections	After every weekly ED simulation during the SDT meeting, the lead for the session (VB or EP or other SDT team member) facilitated a reflective discussion using the EDI reflexivity tool (Fig. 1). When possible, for the first 3 months, the team submitted deidentified field notes in written or audio diary form based on these discussions
Self-reflections	During the first 3 months of tool use, participants were invited to complete structured reflections (Additional file 2) via survey monkey. Participants did not have to answer all the questions in the survey, rather it was a tool that facilitated a reflective process for them to choose to engage with

### Data analysis

VB and EP conducted an inductive thematic analysis of all data sources in Nvivo 12 with BS available for discussion of discrepancies [13]. The initial interviews were analyzed, and results shared with the group, as part of the collective reflective process. Simultaneous data collection and familiarization took place throughout the remainder of the study period. EP and VB met regularly to discuss trends and during these meetings discussed their own reflexivity related to the data. Key trends were shared with the team throughout the study period through ongoing informal discussions and formal education meetings. At the end of the study period, all data (including initial interviews) were analyzed using Braun and Clarke’s six step process [13]. The final manuscript was shared with the team for input. The results presented represent a collaborative and wholistic sense of our teams’ experience with the implementation of this tool.

## Results

We conducted 10 initial interviews and 8 follow-up interviews of the SDT team, two staff members were unavailable for follow up interviews due to maternity leave and annual leave: median (range) duration of initial and follow-up interviews were 25 (18–38) min and 33 (26–60) min respectively. Fourteen self-reflections were completed and field notes from over ten independent uses of the tool were included in the analysis. Throughout this process and during ongoing conversations our team has learned more than can be captured in these results. We aim to summarize key findings that might be useful to the simulation community and for others considering such an approach.

The results section is organized in three sections: (1) tool implementation, (2) impact of the tool, and (3) key themes identified throughout the process. We found that the tool could be successfully implemented. Engaging with the tool had several impacts on SDT team culture, skills, and practices. The process illuminated themes that may resonate with other SDT teams moving towards aligning their programs with EDI.

### Tool implementation

The tool was implementable within our committed team and simulation delivery structure. We did not require any additional time or resources but using it did depend on willingness of the team to participate. Conversations were variable in length with some uses fostering deep discussion and others prompting only brief reflections. Most conversations lasted about 5–10 min. Initially, the SDT used the rigid structure proposed in Fig. 1 but as the conversation was normalized it was more seamlessly integrated into a general SDT debriefing. After initial focused familiarization using the initial tool, the team felt a streamlined approach of combining it with the more general SDT debriefing maintained the integrity of the conversation while making it more likely to be engaged with. Twelve months after initial implementation, the team estimates that EDI reflection is now part of ~ 80% of the SDT post-sim debriefings. The tool has been well-received as demonstrated by the quote below. Some of the early skeptics became the most faithful instigators of the conversation.

“I think it really did create a place of, you know, genuine interest in discussion. At first, I wasn’t quite sure how that discussion would go, or how receptive as a team we would be to it but the ones I was involved in were actually quite positive.”—Participant 10, follow-up interview

### Impacts of the tool

Throughout the course of the study period and in the follow-up interviews we identified several impacts of the tool. Table 2 outlines the impacts of the tool for our SDT culture, skills, and practices. Of note, there were no large-scale changes to our simulation program during this time and there were no specific writes or re-writes to scenarios with EDI learning objectives in mind. Some scenarios were altered for more diverse representation (i.e., gender for case with ST-elevation myocardial infarction changed from male to female). We found that meaningful conversations and reflections related to the relevance of EDI within simulation can occur in the confines of a pre-existing curriculum.

**Table 2:** Impacts of EDI tool on SDT culture, skills, and practices

**Impact**	**Explanation**	**Example**
**Team reflexivity**	The tool effectively prompted the team to examine their own feelings, reactions, motives, sources of power, and make-up and how those may have impacted the simulation design, delivery, or debriefing	“I wasn’t doing the debriefing for that case but we ended up having a bit of a discussion [in the SDT debrief] about why we didn’t go there [into a conversation about gender in the workplace]. It ended up being valuable for our group because it allowed some introspection for us as debriefers first to think about why or why not, we would go there. And maybe I would have been too quick to go there because it’s something that I’m interested in and I think is quite important. But that might not be the same for everybody. So just starting that conversation within ourselves.”—Participant 4, follow up interview “I think it gets people thinking because I don’t think we generally take time to stop and think about it.. you know underlyingly thinking about equity, diversity inclusion, I think it makes you stop and actually think, "Am I doing that? And am I doing it well? Or am I at least making some sort of effort towards changing?”—Participant 2, follow up interview “I am unsure if this [a faculty member asking the SP if he wanted to change his assigned name] was odd, insensitive or actually quite thoughtful. It was awkwardly brushed over and he declined to change. I am not sure how he felt about it, but the conversation made me uncomfortable. Perhaps I should have enquired more.”—anonymous self-reflection
**Normalization of conversations related to EDI among the SDT**	The team identified that regular conversations about topics related to EDI became normalized within the simulation team organizational culture	“It, it’s not really a faux pas at all anymore. It’s just, it’s just part of the conversation now. Which, which it wasn’t before…After any simulation that we do, you could say, "Oh shall we have a chat about EDI?", and everyone would just fall into place and be like, "Yeah, no worries. Let’s talk about it.". Whereas before, that would have been an odd conversation to have.”—Participant 6, follow-up interview
**Building confidence to engage in EDI conversations with sim participants**	Many on the team felt more confident in engaging in debriefing conversations related to EDI topics and reflected on how general simulation debriefing skills translate well into conversations related to EDI. However, many still feel less comfortable than in conversations related to standard teamwork and medical concepts	“I guess I just feel much more confident about naming a dynamic if it was there… If I thought there was some discomfort and some tension, I would be confident to name that as part of my debriefing repertoire, whatever was the source of that, including EDI. I would like to think I would do that in the same spirit as most things, which is, “Tell us how you’re thinking, tell us if we’ve got this wrong somehow?”, not with the idea of telling people how they should manage it.”—Participant 7, follow-up interview “I suppose the summary [of the impact] would be it’s just engaging in conversations I would most likely previously have avoided.”—Participant 6, follow-up interview “That kind of degree of trepidation, at least for me, it has not kind of fully not at all resolved in this seven month period, like at the beginning all of the interviews and I think discussions we were having a group there’s a lot of fear around that specifically. And I think we maybe have a bit more insight into that fear now and can couch it but definitely has not been resolved”—Participant 1, follow-up interview
**Highlighting complexity**	Conversation prompted by the tool started to unravel the complexities of EDI in simulation. The team began to wrestle with, but rarely reconciled, the trade-offs and impacts of decisions related to design, delivery, and debriefing	“Where’s the line between what is a case that you have to do that [get input from a relevant group] for and a case that you don’t? It probably changes for everybody. So, if I designed a paediatrics simulation with parents who play an important role in that simulation, but I’m not a parent—do I have to reach out to a parent to inform that perspective and design of the case?…It probably depends on what the learning objectives are, and how dependent that is on the scenario. So…it starts to get a little bit complicated when you when you think of it all. You can kind of understand why people’s reaction is oh, well, we actually just won’t do that. Because it sounds hard.”—Participant 4, follow-up interview
**Value signposting**	The implementation of the tool effectively signaled to the SDT that EDI is an important value of the simulation service	“There is a certain sort of sense of almost like duty that this something we’re supposed to do. So whereas before you could be completely oblivious to it [EDI]…now, not addressing it, I think would feel like a failure. So there’s increasing confidence by doing it but it’s almost now an expectation.”—Participant 6 follow-up interview “I guess it is a valuable cultural tool in terms of signalling the importance of EDI issues in a team. If all the leadership in medical education, wherever I was, were on board, I think that would just say something really powerful about the value that’s placed on EDI.”—Participant 8, follow-up interview
**Team familiarity**	The tool was a vehicle for the SDT to engage in meaningful conversations with each other which led to deeper understanding of colleagues	“The opportunity to have actual in depth, real conversations with my colleagues that are not just superficial is powerful. I have learned things about my colleagues that I didn’t know, that has actually been good, but also sometimes a little bit confronting. I think they have actually allowed us to grow more as a team and become a little bit closer and more understanding of where everybody’s at.”—Participant 4, follow-up interview
**Marginal changes**	There were no large-scale changes or new programs introduced. SDT members described some small changes such as patient names and demographics, more frequently engaging in an “Acknowledgement of Country”, and many subtle choices related to debriefing topics. These changes were marginal and hard to measure. These changes were at times challenging for the SDT team to appreciate	“We made an effort to be aware of the diversity and inclusion more prominently, during our simulations from giving an acknowledgement of country…and then also, just being aware in terms of points we might be noticing from different positions in the team, or interactions between a family or between a patient and the team. I think we tried to be more aware of those issues in our debriefing topics that we chose to discuss.”—Participant 8, follow-up interview
**Translation into Clinical Practice**	SDT members agreed that simulation is a place where signalling and role-modeling around EDI within the organization is occurring. Some members of the SDT in clinical roles felt that the tool enhanced their understanding and approach to EDI, not only in simulation but also within the clinical workspace	“I think that the language that makes up how I think what we will be doing is essentially role modeling how we would expect people to broach the topic, and how we might tactfully and safely in a psychologically safe way. Raise the potential that people may have experienced some degree of bias or stereotyping in a simulation will serve as an excellent role model for people who need, or who ought to raise similar concerns in a clinical environment.”—Participant 1, initial interview “I think what it has changed for me a lot is the way that I approach diverse patient groups on the floor. And it has allowed me to have a lot more open questioning, and then also a lot more understanding for my teams on the floor.”—Participant 5, follow-up interview

### Key themes

In initial interviews we identified three main themes: (1) individual and team growth, (2) fear of “getting it wrong”, and (3) tension between bias towards action and need for slow reflection. Throughout the study period, there was an evolution of thinking around these three themes, and they remained prominent in reflections and the follow-up interviews.

#### Individual and team growth

The overwhelming sense from follow-up interviews was that individuals, and the SDT collectively, started a journey. The initial interviews underscored uncertainty as it relates to understanding and attitudes related to EDI. At baseline, the SDT did not have a universal understanding of the specific concepts and held various, sometimes conflicting, views about the relevance EDI to our simulation work. Throughout the year, some uncertainty about the specifics of EDI concepts resolved and some persisted. There was, however, a clearer shared understanding within the SDT team about its relevance in simulation design, delivery, and debriefing. There was a palpable desire from the SDT to learn more and continuously improve.

“I just felt so ignorant. For me, it was a real blind spot. And as a result, obviously, when you do feel really ignorant about something you think a bit more about it to try and bridge some of that gap.” —Participant 5, follow-up interview

“I decided I would actually take it seriously and try and reflect on it, and have been somewhat surprised by the sort of growth or journey or sort of thing that, we’ve been on.”—Participant 6, follow-up interview

One of the most obvious shifts for the team was a broadened understanding of the concepts. Initial interviews often fixated on race and language but throughout the study period the team reflected on how other aspects of diversity (i.e., gender, profession, age, disability, sexual-orientation, illness portrayal) also impact EDI in simulation. This widened lens facilitated entry into conversations about the principles of EDI, particularly for those who were less confident navigating conversations. Many took comfort in realizing that the principals of debriefing provide a sound scaffolding for leading conversations related to EDI but confidence about doing so remained an issue for our group.

“I actually think that general principles that debriefing is built upon are really sound for any conversations that you’re having. And I think as long as you’re not trying to tell people how they should manage their EDI issues in clinical care, then you’re probably going to have a good conversation. And maybe though, we need to be supporting facilitators to be willing to have some conversations that they might not feel entirely comfortable.”—Participant 7, Follow-up interview

A critical evolution for our SDT was a gradual movement from understanding that EDI is not something that we can just “know” and “do” and “fix”, towards the realization that they are concepts that we must constantly negotiate and be mindful of.

“I’m also comfortable saying that maybe I didn’t have very good knowledge, but probably also still don’t. I think that was a a mental shift for me to rather than assuming that, either I knew or someone else knew the answer that maybe it’s just the journey. I feel like the first step is acknowledging that there probably is a bit of a problem. And then, I think we’re trying to feel our way through it, on a bit of a journey.”—Participant 6, follow-up interview

#### Fear of “getting it wrong”

There was a persistent, ongoing, and sometimes paralysing fear of “getting it wrong”. The ‘it’ in “getting it wrong” meant different things to different people at different times. The predominant fear was of tokenism. The fear of making a symbolic but empty effort at interfacing with EDI was mentioned in nearly every initial and follow-up interview and was a frequent topic of informal conversation for the group. Many participants worried that any deliberate attempts to incorporate EDI within the simulation design could become a “tick box” exercise without meaningful associated outcomes. Other ways in which participants were worried about “getting it wrong” are outlined in Table 3. The team did not identify clear solutions to these problems and is starting to recognize that there are not any. Rather, we have attempted to continue to address fears through ongoing discussion and reflection as a group.

**Table 3:** Fears related to navigating EDI in simulation

**Fear**	**Explanation**	**Evidence**
**Tokenism**	The team was concerned about worried about empty symbolic nods to EDI that did not have meaningful impact	“I don’t really think there’s been very much design change. I suspect, there’s probably some roads that we could improve on with design, but also, I think it’s potentially one of the most difficult and or dangerous things to try and change because there’s certainly a risk of being tokenistic.”—Participant 6, Follow-up interview
**Stereotyping**	The team was concerned about the risks of stereotyping patients in design and delivery of simulation. They reflected on the powerful signalling that simulation sends and were concerned about the problems associated with negatively or inaccurately portraying a group. Most participants had problematic experiences they could draw on that they feared recreating. The team reflected however, that we do rely on some form of stereotyping in the portrayal of all our patients and that navigating appropriate and inappropriate representation is a complicated issue	“[I worry about] stereotyping and also then having conversations that about people without people. And also getting into generalizations when specifics matter about populations or groups and just saying things like, ‘they always don’t want to make eye contact and other such shit things that we’ve heard about groups’… I do think most people are trying to do something good. But that isn’t good enough.”—Participant 7, Follow-up interview “[recalling a scenario during training] then the case started and the mannequin’s voice was basically the simulation tech in a Jamaican accent and this person had been found down outside of this supervised consumption site and was in torsades, because their methadone interacted in prolonging their QTc. So they basically played into every damaging, problematic stereotype that one could imagine with the black mannequin being the first time that any of us had seen it. Everybody in the room felt extremely uncomfortable”–Participant 4, Follow-up interview “We would never in a million years, have someone play a black person who wasn’t black. And yet it occurs to me that we have simulated patients playing dementia patients every month. And it seems that old people, for some reason it’s fine to appropriate their experience… I don’t know what the answer is.”—Participant 7, Follow-up interview
**Being Offensive**	Participants feared causing offense to populations as well as participants in both simulation design, delivery, and debriefing. This fear of being offensive likely underpins why some topics felt easier to broach while others more challenging	“I think that’s what, for me, can be a little uncomfortable when you walk into territory where you have the potential to cause offence to people, because you don’t, you know, you don’t want people to walk away from the simulation, either a) offended by what was said, or what was discussed, or how a case was portrayed, or b) Walk away with a negative experience.”—Participant 10, Follow-up interview “I think people find themselves addressing issues related to diversity, equity & inclusion, that they feel on safe ground with, whether that be because they have a personal experience or a personal lens, or something that gives them some credibility, I think is probably a big worry for people. I think if I had to boil it down to one, worry, that would be it, would be credibility.”—Participant 4, Follow-up Interview
**Maintaining Psychological Safety**	Participants recognized that psychological safety might be threatened by challenging conversations and that having such conversations could be harmful with a group that does not feel safe. At the same time, they recognized that if psychological safety has been appropriately fostered, simulation could be the perfect place to have difficult discussions. Participants realized that different debriefers will have varying skill and comfort level in navigating this delicate balance	“You know, I guess you get a bit uncomfortable, because they’re, you know, they are potentially some high stakes conversations. But I think for me, it’s just about being very explicit that we can we come at this from a place of, of learning and practice improvement?” —Participant 10, Follow-up interview “And then the thing is that with any kind of scenario, it all depends on who’s going to be the participants and who’s going to be debriefing, as well. Because if you’ve got someone that may not necessarily have the debriefing skills to bring it up in a sensitive way, there is a lot of potential to do harm as well.”—Participant 5, Follow-up interview “It sounds like we’re a little bit fearful that that might threaten psychological safety but at the same token, wouldn’t it be great to be able to discuss and reflect on those feelings of discomfort in a place that I think we’ve tried very hard to make really safe”—Participant 9, Follow-up interview
**Signal-to-noise mismatches**	Participants worried that some attempts to increase diversity and representation may detract from the “medical learning” or objectives for the case as learners inappropriately focus on those cues. They discussed this problem s diagnostic of a broader issue within education but also found that navigating the real considerations it presents a challenge	“There’s this sense that if you mix things up a little bit like make the patient demographics or the situation, more culturally or ethnically variable, that sometimes that would seem to be a bit like noise for the participants, like they would start focusing on why that is and looking for another message or agenda.”—Participant 10, Follow-up interview
**Sim as the appropriate method**	Participants, while acknowledging that EDI was relevant to simulation, worried that it may not always be the right forum to specifically approach learning objectives specifically related to EDI. Matching learning objectives with appropriate modality was highlighted as an ongoing concern for educators	“We risk not approaching or talking about these things but it could be that the way that we actually do it needs to be different…simulation may not actually be the right modality. To do that, it could be that some case based conversations are actually a more powerful way.”—Participant 4, Follow-up interview
**Social Implications**	Some participants feared that attempts to advance EDI in simulation may be negatively perceived by those outside of education circles	“I think one fear that I had was that… I don’t know if you’ve ever had, how much you have encountered this in your career, but a lot of the time people within the education circle are very positive, very happy. They describe how things can be done very well and then a lot of your ED colleagues are probably FACEMs who have been around for 20 or 30 years who most of them are white, grumpy old men who just roll their eyes and have absolutely no interest in being involved in any of this.”—Participant 6, Follow-up interview
**Not doing enough**	While participants all sorts of fears about acting, they also had fears about doing too little. There was a recognition among the group that not addressing EDI in simulation is also a harmful path	“I think we can’t do nothing, I think we need to do something and it’s a really important topic. I think it’d be an ongoing challenge but I think starting out on the journey to explore that is essential and I think it’s an option not to also be the classic archaic medical profession”—Participant 6, Initial interview “I like to think that my own awareness translates into better design, delivery and debriefing. But I am worried that that’s also not quite enough. But don’t know exactly how to get to that next step…I also debate in my mind about whether that’s necessary. I kind of think actually running a simulation program, that is aware of issues related to equity, diversity and inclusion, and is mindful of them and is at times including that a little bit in design and delivery and, you know, at times bringing that in debriefing is actually okay, if that’s not the front and centre objective of what you’re doing. So, I go back and forth in my mind about whether we need to be doing a lot more than we currently are or whether what we’re doing isn’t enough? I don’t really have the answers to that.”—Participant 4, Follow-up interview

#### Tension between bias towards action and need for slow reflection

There was constant negotiation between desire for action and the need for reflection. Initial suggestions from the team often enthusiastically focused on designing new simulations for specific diverse patient populations. These suggestions, while well-intentioned, sometimes came without clear consideration of the risks or clarity around why simulation should be used. One team member described the challenges of helping the team slow down.

“What I find challenging is everybody’s brought ideas about sim design. I’m trying to be sort of supportive, but at the same time, sometimes, I feel like some of those suggestions are not very good ones and often the source of them is enthusiasm. But I think the lack of mature reflection on the idea and having to say no, let’s not just tweak something, for instance… that’s probably my challenge, is thinking about, how do we temper people’s ‘white urgency’”—Anonymous self-reflection

While reflection prevented some potentially problematic manifestations of EDI in simulation, there was an ongoing desire to “do something”. Throughout the study period there were no events with specific EDI learning objectives added, rather continuous restrained shifts to our everyday simulation based on collective reflection. Changes—such as incorporating an Acknowledgement of Country, entering debriefing conversations that may have previously been avoided, diversity in names and backgrounds of SPs, and being more attuned to power dynamics—were more subtle than the team expected at the outset.

“I kind of thought we, you know, we do this project, we identify some things that we can do better, and then we would just kind of do them better. There’s actually a lot, I have found it to be a lot more complicated than that.”—Participant 4, follow up interview

As a team, we have not yet, and likely will not, perfectly reconcile the need to act with the need to carefully reflect on ourselves and our approach.

## Discussion

**Fig. 2:**
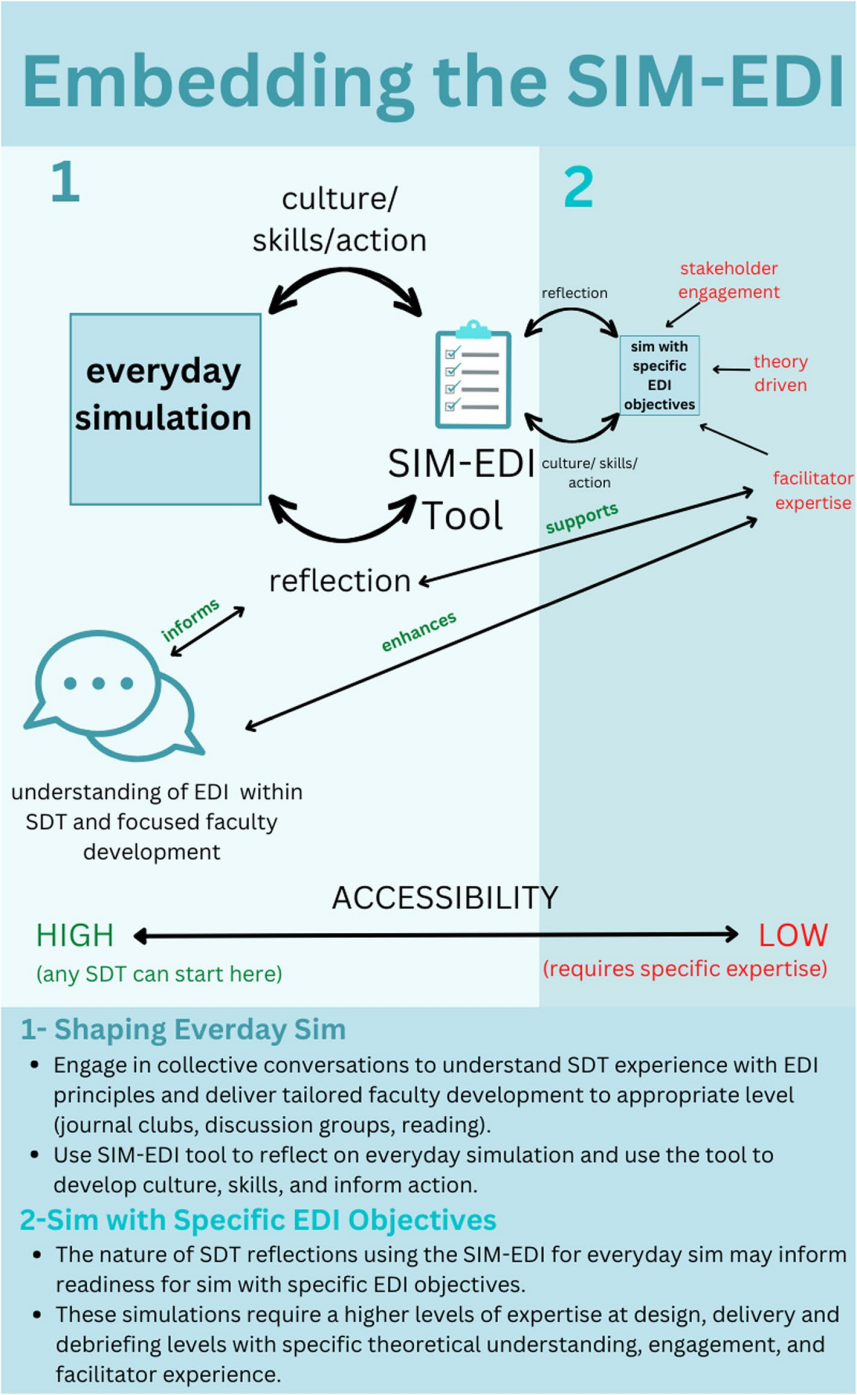
Embedding the SIM-EDI

We present our SDT’s experience using the SIM-EDI tool which we found enhanced our team’s reflexivity in relation to EDI. The specific outcomes of our team’s experience will be unique but the tool’s utility as a practical link between ideals and actual practice is likely to be generalizable to other groups. Our main practical suggestion for teams seeking to foster EDI in simulation is to use the SIM-EDI tool to simultaneously understand and shape your SDTs approach to everyday simulation. This can be even more effective when paired with collective conversations and targeted faculty development (Fig. 2).

Fundamental to adopting this approach to EDI is an understanding that being inclusive, equitable, and diverse is a never-ending journey for facilitators. It requires continuous self-education, reflection, and interrogation of practices and assumptions. There is no quick solution or simple intervention but rather an ongoing and constant negotiation. The SIM-EDI tool shapes everyday simulations and has facilitated deeper understanding of our readiness for simulation with specific EDI learning objectives.

### Shaping everyday simulation

Use of the SIM-EDI tool has cemented the understanding within our SDT that EDI is relevant to every single simulation session we facilitate. Previous research has shown simulation to be a moment of cultural compression—a key time when values, beliefs and practices are transferred—which underscores the importance of care and attention to EDI in our everyday work [14]. The tool has highlighted to our team that the design, delivery, and debriefing choices that we make every day are both impacted by how we see the world and go on to shape how others do too. Figure 2 highlights that groups can be meaningfully attending to EDI in everyday simulation without the need to design new events with specific EDI related objectives.

The SIM-EDI prompted the conversations and reflections that led us to seek out literature and advice related to a variety of topics relevant to everyday simulation, which we see as a success at its ability to promote our ongoing development. For example, conversations prompted by the SIM-EDI led us to find literature related to simulation design, delivery, and debriefing. We found that Craig et al. recently published a focused checklist for *designing* equitable and diverse simulations [15]. Others have highlighted ongoing challenges associated with *delivering* simulation because of a lack of diversity of mannekins and considerations related to safety of simulated participants [16, 17]. Meanwhile others have drawn attention to that reality that the *debriefing* phase is impacted by culture and facilitator experience. (18) Such resources bolstered our understanding, shaped our approach, and inspired ongoing debate within our group. We anticipate that the tool will also help your teams identify relevant questions and inspire you to find answers about EDI in everyday simulation in a way that promotes continued growth of individuals and the program.

We also found that the SIM-EDI allowed our team to reflect on subtle intersections of EDI every session. We found ourselves asking, “who held conversational power in the room?” or “why didn’t I enter into that conversation about gender when the moment came?”. These discussions may not have resulted in easy to count outcomes but did have measurable impact on values and attitudes (Table 2). We are confident that the tool has gently shaped our everyday simulation towards the ideals of EDI but with plenty of more work for us to do. We hope that other teams might have a similar experience.

### Designing simulation with specific EDI learning objectives

Early in this process of collective reflection we found ourselves grappling with the fact that we did not have any simulation events with specific EDI learning objectives. Afterall, the literature is teeming with examples of uses of simulation for EDI specific learning (i.e., implicit bias training, cultural training, population specific considerations) [18–21]. We found ourselves asking, *“Should we be doing something similar?”* The intrinsic desire to “do something” was only just overpowered by our fears outlined in Table 3. These fears (risk of stereotyping, tokenism, matching appropriate educational methods to objectives etc.) continue to slow us down—and we think appropriately so. A recent review article of simulation for exploring culture highlights significant problems with many of the approaches taken [18]. A lack of theoretical underpinning, risk of stereotyping and, lack of authentic partnership are just a few of the key issues raised. There is significant and real risk in “getting it wrong”. Like us, many SDTs may not have the in-house expertise and resources to thoughtfully coordinate simulation with specific EDI learning objectives. Figure 2 shows how the SIM-EDI tool can help inform a reflection on local readiness for designing and delivering simulation with specific EDI objectives and highlights that additional expertise may be required to do so.

Conversations prompted by the SIM-EDI tool helped our group understand our potential deficits as it relates to delivering simulation with EDI specific learning objectives (expertise, resources). The tool prompted us to look towards groups who are leading the way to offer guidance about how to “get it right”. In their recent article “Equity, diversity, and inclusion in simulation” Nakajima et al. give thoughtful suggestions on how EDI can be embedded in simulation design, delivery, and debriefing [4]. Similarly, Vora et al. offer recommendations for the use of simulation to address structural racism and implicit bias [22]. Both highlight the need for aligning modality with objectives, ensuring authentic engagement of populations in design, mitigating risks of stereotyping in delivery, and confidence in conversational skills. They also highlight the critical nature of reflexivity for those involved in design and facilitation. Reading these articles, we were somewhat overwhelmed by all the care and attention it takes to “get it right”. With our group and within our context, we are not currently set-up to do so. Our regular use of the SIM-EDI tool has contributed to informing this realistic understanding of readiness. It has prompted us to slow down. At the same time, we are confident that regular conversations using the SIM-EDI tool will help us grow as individuals and as a program to the point where we are better positioned and able to prioritize simulation with specific EDI learning objectives.

Our experience highlights that the SIM-EDI tool is not designed as a recipe for EDI in specific simulation sessions. It is an enabler of honest reflection and a prompt for ongoing development.

## Limitations

This study was conducted with a SDT that was willing to engage in conversations related to EDI in our simulation work. While our team existed on a spectrum of understanding of principals and values related to EDI, an important commonality was a willingness embody a growth mindset as we engaged in conversations together. This reality highlights the reality that this tool may best used by teams who have high levels of trust and readiness to engage.

Another limitation of the SIM-ED tool is the potential creation of an echo chamber. SDTs may share similar world views which could limit the benefits of collective reflection. We found that discussions often led us to searching and sharing new information, but this might not always be the case. Deliberate efforts to pair the use of the tool with other sources of learning for the group such as a book/journal club/expert speakers may be useful.

The main limitation of the research project is that field notes were only collected on 10 uses of the tool despite it being dozens of times. Practicalities prevented this from happening. It is possible that we would have been better able to share more actionable changes with more robust data collection. Furthermore, the nature of the collaborative autoethnography means that data was collected and interpreted by our team. The potential for social desirability bias impacting our findings exists. We attempted to mitigate this by including an outsider, BS, in the data collection and analysis process.

## Future opportunities

The experience of the SIM-EDI tool within our group opens the door for numerous other uses. We plan to expand the use of the tool beyond the ED and embed it within regular practice for the Gold Coast Hospital and Health Service Simulation Service. Furthermore, the tool should be adapted and studied outside of our local context. The potential to adapt the tool for clinical use, to prompt reflexivity for clinicians in the care-delivery context, is also an exciting avenue of future application and research.

## Conclusion

The SIM-EDI tool can effectively promote reflexivity among faculty in an emergency department simulation program. The tool can be implemented in a team with appropriate readiness, it impacts attitudes and behaviours, and facilitates individual and team growth.

## Supplementary Information

**Additional file 1.** Semi Structured Interview Guide.

**Additional file 2.** Email Reminder for Self-Reflection and Self-Reflection Tool.

## Data Availability

The dataset generated is not publicly available to maintain participant confidentiality but parts of the data may be available upon reasonable request.

## Abbreviations

SIM-EDI tool: Simulation equity, diversity and inclusion tool
EDI: Equity, diversity and inclusion
SDT: Simulation delivery team
ED: Emergency department
LGBTQ2 +: Lesbian, gay, bisexual, transgender, queer and two-spirited
