# Supplementary material for: Exploring equity, diversity, and inclusion in a simulation program using the SIM-EDI tool: the impact of a reflexive tool for simulation educators
Source: Adv Simul (Lond). 2023 Mar 31;8:11. doi: 10.1186/s41077-023-00250-7 (PMC10067255; doi:10.1186/s41077-023-00250-7)
Supplement: Supplementary file 1 — Additional file 1. Semi Structured Interview Guide. [file 41077_2023_250_MOESM1_ESM.docx]

Additional file 1 – Semi Structured Interview Guide

Thank you for agreeing to participate in this interview. As a reminder it will take 15-20 minutes and will be audio-recorded then transcribed. Within the next two weeks you can get in touch with me to review your transcript if you wish. Today we will be talking about equity, diversity, and inclusion in simulation. You can feel free to skip over any questions you do not wish to answer.

Introduction questions

- Tell me a little bit about yourself and your role on the simulation team
- Since we are talking about equity, diversity, and inclusion today can you tell me about what those concepts mean to you.
- How did you learn about these ideas and concepts
  - Explore formal training/in clinical training/personal reading etc.

Current state

- Right now, describe how/if equity, diversity, and inclusion is incorporated into simulation in the ED…
  - Design
  - Delivery
  - Debriefing
- Tell me about the last simulation you were at where issues related to equity/diversity/inclusion were relevant
  - Explore planned vs unplanned
  - Types of themes that came up

Problems

- Have you ever been involved in a situation related to equity/diversity/inclusion in simulation that has made you feel uncomfortable?
  - Can you describe it for me?
- What do you find most challenging as a simulation facilitator when it comes to equity, diversity and inclusion?
  - Why do you think that makes you uncomfortable/what makes that hard?

Ideal state

- In an ideal world how do you think the simulation team would approach equity, diversity, and inclusion in ED sims?
- What is preventing us from doing that?

Experience with structured reflection (only to be used in second set of interviews)

- For the past few months the simulation team has been engaging in structured reflection after cases – tell me about that process?
- Describe how those conversations impacted you as a simulation facilitator?
- Were there any meaningful changes you noticed?
- How would you adapt this tool for future use?
- If you were leading a simulation moving forward would you use it? Why/why not?

Future

- What steps do you think the simulation service should do to enhance equity, diversity, and inclusion in simulation design, delivery and debriefing?
- If you could change one thing, what would it be?

Anything else you think I should know?
