# Supplementary material for: Exploring equity, diversity, and inclusion in a simulation program using the SIM-EDI tool: the impact of a reflexive tool for simulation educators
Source: Adv Simul (Lond). 2023 Mar 31;8:11. doi: 10.1186/s41077-023-00250-7 (PMC10067255; doi:10.1186/s41077-023-00250-7)
Supplement: Supplementary file 2 — Additional file 2. Email Reminder for Self-Reflection and Self-Reflection Tool. [file 41077_2023_250_MOESM2_ESM.docx]

Additional file 2– Email Reminder for Self-Reflection and Self-Reflection Tool

*Email*

Dear Participant,

This is your reminder email to engage in self-reflection related to your experience with EDI as a simulation facilitator.

You can access the self-reflection tool here [insert link]

A reminder that completing this reflection is voluntary and is not linked to your employment. Your responses will remain anonymous.

If you have any questions please contact Eve Purdy at [eve.purdy@health.qld.gov.au](mailto:eve.purdy@health.qld.gov.au).

Thank you for participating.

Eve Purdy

Please note: These responses will be submitted via SurveyMonkey, which is based in the United States of America. Information you provide in this form, including any personal information, will be transferred to SurveyMonkey’s server in the United States of America. By completing this form, you agree to this transfer. The collection, use and disclosure of your personal information will be subject to the privacy laws of the United States of America as well as SurveyMonkey’s privacy policy. You should consult the SurveyMonkey privacy policy for more details, which can be found at <https://www.surveymonkey.com/mp/policy/privacy-policy/>

*Self-Reflection Tool* (Week 1)

There are a number of prompts below – please pick **two** or **three** that you are most keen to reflect on. These are meant to be a starting point for you to share your thoughts and there are no correct or incorrect answers.

Please write your unique code (first letter of your favourite food, favourite number, favourite colour – ex T3G for taco, three, green) _______

1. Describe a recent time in simulation when you felt issues related to equity, diversity, and inclusion were incorporated well.
2. Describe a recent time in simulation when you felt there might have been harm related to equity, diversity, and inclusion.
3. What do you find most challenging when considering how to incorporate equity, diversity and inclusion in simulation?
4. Describe any fears you have about incorporating equity, diversity, and inclusion into simulation.
5. As simulation facilitators there is a power imbalance with simulation participants. How does awareness of that power impact your approach? What other sources of power do you hold?
6. Find three resources related to EDI in simulation or education. Share with us why you found them useful for your job as a simulation facilitator.
7. Share any reflections or ideas for the simulation team to better incorporate equity diversity, and inclusion into our simulation approach.
8. Any further thoughts or reflections _____

*Self-Reflection Tool* (All other weeks)

There are a number of prompts below – please pick **two** or **three** that resonate most with your experience this month and what you are most keen to reflect on. They can be different prompts than you engaged with the last time. They are meant to be a starting point for you to share your thoughts and there are no correct or incorrect answers.

Please write your unique code (first letter of your favourite food, favourite number, favourite colour – ex T3G for taco, three, green) _______

1. This month did any topics related to equity, diversity or inclusion come up during the simulation debriefings? If so, describe…what worked? What could have gone better?
2. Were there any times that you felt uncomfortable (or avoided) navigating a conversation related equity, diversity, and inclusion? Why do you think it was challenging for you?
3. Regarding the most recent simulation you facilitated, describe the biases you may have brought into the scenario design, delivery, and debriefing? What impact might these biases have?
4. What you do plan to do next month to incorporate equity, diversity, and inclusion more meaningfully into your simulation design, delivery and debriefing? What can the simulation team do to support you in those aspirations?
5. Find three resources related to EDI in simulation or education. Share with us why you found them useful for your job as a simulation facilitator.
6. Describe the last simulation team debriefing after the session using the reflective tool. What was helpful? How could those discussions be more useful?
7. Any further thoughts or reflections _____
